# Pharmacotherapy Literacy of Parents in the Rural and Urban Areas of Serbia—Are There Any Differences?

**DOI:** 10.3390/medicina55090590

**Published:** 2019-09-13

**Authors:** Dušanka Krajnović, Stana Ubavić, Nataša Bogavac-Stanojević

**Affiliations:** 1 Department of Social Pharmacy and Pharmaceutical Legislation, Faculty of Pharmacy, University of Belgrade, 11221 Belgrade, Serbia; 2 Medicines and Medical Devices Agency of Serbia (ALIMS), 11221 Belgrade, Serbia; stana.ubavic@alims.gov.rs; 3 Department of Medical Biochemistry, Faculty of Pharmacy, University of Belgrade, 11221 Belgrade, Serbia; naca@pharmacy.bg.ac.rs

**Keywords:** parents, pharmacotherapy literacy, rural, pre-school children, pharmacist

## Abstract

*Background and objectives*: Pharmacotherapy literacy (PHTL) is an individual’s capacity to obtain, evaluate, calculate, and comprehend basic information about pharmacotherapy and pharmacy-related services necessary to make appropriate medication-related decisions, regardless of the mode of content delivery (e.g., written, oral, visual images and symbols). It is already proven that low PHTL of parents can cause serious problems in the treatment of a pediatric population. We aimed to identify the differences in parental PHTL levels, socio-demographic and health-related characteristics (chronic disease of a child, breastfeeding of a child, annual visits to a pediatrician, parental-self-estimation of health status) between rural and urban areas and to investigate the influence of living in rural areas on a low PHTL level. *Materials and methods*: Our study was cross-sectional with a validated 14-item instrument (“Parental pharmacotherapy literacy questionnaire—Serbian”), which assessed overall PHTL and its three domains of knowledge, understanding and numerical skills necessary for the safe use of medicines. We analyzed 250 parents of pre-school children (1–7 years old) in rural areas and 182 parents from urban areas in Serbia. *Results:* Every tenth parent from rural and every fourth parent from urban areas had the highest PHTL level or more than 85% correct answers. However, 51% and 28% of parents in rural and urban areas, respectively, had a low PHTL level (less than 65% correct answers), [Х^2^(1, *n* = 432) = 33.2; *p* < 0.001]. Parents from different areas statistically differed in age, education level, employment, breastfeeding and annual visits to pediatrician rate. Those from rural areas had almost twice the probability of low PHTL levels (ORa = 2.033; *p* = 0.003) than their urban counterparts, independently of other examined parental characteristics. *Conclusions*: Parents from rural areas have more difficulties to obtain, evaluate, calculate and comprehend basic information related to pharmacotherapy than parents from urban areas.

## 1. Introduction

Health literacy has become one of the most important issues in European policy and it is recognized as an important component of health care [1]. It refers to “the degree to which individuals have the capacity to obtain, process, and understand basic health information and services needed to make appropriate health decisions” [2]. In the World Health Organization’s Shanghai Declaration on Promoting Health, health literacy is seen as a global issue and one of three key parts of sustainable development [3]. In Serbia, similar to eight other European countries, it is estimated that on average every second person who participated in a survey were inadequately health literate or marginally health illiterate, as measured by the S-TOFHLA (Short Test of Functional Health Literacy in Adults) instrument [4,5]. In a U.S. nationally representative study, Yin et al. report that almost 30% of U.S. parents have difficulty in understanding and using health-related information [6]. Appropriate literacy, numeracy and knowledge skills are essential for the comprehension of health information and rational medicine use [7].

Although it is well known that inadequate levels of subjects’ health literacy may negatively affect pharmacotherapy outcomes and safety of care delivery, Raynor stressed that the priority for pharmacists was to address the low literacy in terms of medication, particularly in areas of greater social deprivation and where significant health inequalities exist [8,9]. The definition provided by Pouilot et al. [10,11] referring to the medication literacy, is very similar to the pharmacotherapy literacy (PHTL), which could be defined as an “individual’s capacity to obtain, evaluate, calculate, and comprehend basic information about pharmacotherapy and pharmacy related services necessary to make appropriate medication-related decisions, regardless of the mode of dissemination (e.g., written, oral, visual images and symbols)” [12]. Numerous studies have highlighted that difficulties in the interpretation of information about medicines are very common in all populations, especially among parents [13,14]. Moreover, populations from a rural area have lower functional health literacy, which is the ability to apply literacy skills to health-related materials [1,2], compared with an urban population [15]. Current data are addressing the existing inequalities between rural and urban populations in access to health information as well as health literacy [16,17].

Many published studies have demonstrated that health literacy levels are significantly associated with socio-demographic and health data [18,19,20]. Researchers have also stressed the differences between child mortality in illiterate rural populations compared to urban populations [21]. A recent study from China also showed statistically significant differences in health literacy according to general characteristics: age, education, disabilities in reading and writing, marital status, living status, occupation, monthly income and subjective health status in a rural population [22]. Furthermore, in a study from Scotland, the majority of respondents who reported that they did not have a convenient source of medicines, regardless of urban/rural classification, lived further away from a hospital or a pharmacy [23,24]. Patients from rural populations in Ghana were more guided to source information on medicines in community pharmacies compared to urban population patients [25]. In our recent study [8,13] from Serbia, it was found that limitations in understanding common information about the use of medicines were very often among parents of pre-school children, while practices of medicine use were associated with levels of parental PHTL. However, our studies were performed in the urban areas of the city of Belgrade, without data on PHTL of parents living in rural conditions [8,13]. Moreover, an increase in self-medication in all populations, especially among the young has been observed in the last few decades [26]. As the level of parental PHTL in rural areas could be the potential for future pharmacist interventions, our aims were to identify socio-demographic and health-related characteristics (chronic disease of a child, breastfeeding of the first child, annual visits to a pediatrician, parental-self-estimation of health status) which differ between parents living in rural areas in Serbia in comparison to parents living in urban areas, to investigate the PHTL among these groups and the influence of living in rural areas on low PHTL.

## 2. Materials and Methods

This was a cross-sectional, quantitative and descriptive study conducted in one urban and one rural area in Serbia. Based on the data from 2018, Serbia has 56% of an urban population, which generally refers to the population inhabiting areas that have a greater population density than rural areas. Participants from urban areas were from the municipality of Novi Beograd and from rural areas we surveyed parents from the Municipality of Koceljeva in the Macva district. Macva district has several municipalities, and we chose one that had less than half of the inhabitants in rural areas, which, according to the official national statistics records, is considered to be a typical rural area of Serbia [27,28]. The municipality of Novi Beograd was chosen out of 10 city municipalities as one with the highest income per month and with the largest number of children. We conducted a study on 582 parents. Because our study achieved a response rate of 75%, the total number of analyzed study participants was 432. The study power was calculated based on pharmacotherapy literacy (PHTL) level differences between urban and rural areas. Study power was 95% for type I error equal to 5%. For PHTL level measurements, we used a validated Parental Pharmacotherapy Literacy Questionnaire—Serbian (PTHL-SR) as a perception-based measure with 14 questions [27]. Some of the questions included graphic presentations of a measuring device for the dosage of liquid pediatric medicines and a graphic presentation of packaging for medicines. It was selected for its demonstrated ease of use, reliability, and validity, and also for its suitability in application to different settings, outside the healthcare system, and to different groups of respondents, including the general public, caregivers and parents [8,29]. Questions in the PTHL-SR questionnaire encompassed domains of pharmacotherapy literacy: knowledge, understanding, numeracy and access to medicine-related information.

As was done in our previous study [13], we calculated the total PHTL score and we divided total scores in PHTL into three groups according to terciles. Parents with total scores less than 9 (less than 65% correct answers) were classified in a group with low PHTL level while parents with correct answers between 65% and 85% (total score between 9 and 11) were selected in a group with medium PHTL levels. If parents had more than 85% correct answers (higher than 11 total score) they formed a group with high PHTL levels. Furthermore, we used another 12-item questionnaire to collect some socio-demographic characteristics and the health-related parental and children characteristics. 

### 2.1. Data Collection

Data were collected from May to June 2019 in kindergartens. The survey was performed in five kindergartens in the municipality of Novi Beograd (urban area), chosen on a random basis as well as one kindergarten in the municipality of Koceljeva (rural area). Ethics approval for this research was given by the Committee for Biomedical Research, Faculty of Pharmacy, Belgrade (638/2, 13 May 2019).

The survey instrument was distributed to parents at regularly scheduled parent-teacher meetings in the kindergarten and by interviewers who were trained to describe necessary information about the survey and research. They explained to the participants that they could quit the survey if they were unable to fill out the questionnaire, or if they did not wish to complete the survey. Participants in the survey did not receive any financial compensation for conducting the survey. The inclusion criteria were: at least 18 years old and parent (mother or father) who speaks the Serbian language. Caregivers or legal representatives were considered as parents as well. Individuals with vision problems, those who felt sick or unwilling to participate, persons with difficulties in reading and writing, and anyone with an education in healthcare, were not enrolled in the research. 

### 2.2. Statistical Analysis

The difference between groups of categorical variables (age, gender, education, marital status, employment, smoking status, breastfeeding, self-estimation of health status, number of annual visits to a physician and chronic disease of a child) was examined by the chi-squared (X^2^) test of independence. Data normality for continuous variables was tested by the Kolmogorov–Smirnov test. Knowledge, understanding of information, numeracy skills and total PHTL levels showed skewed distributions and they were compared between the groups by Mann–Whitney U test. Univariate binary logistic regression analysis was used to determine the predictive ability of living in a rural area on low PHTL levels. In order to test associations between the predictor variable (living in rural area) with low PHTL levels independent of confounding variables (socio-demographic and health- related variables) multiple logistic regression was employed. Dependent or PHTL level variables were dichotomized. A low PHTL level (less than 65% correct answers) was coded 1 and medium and high PHTL levels (more than 65% correct answers) were coded 0. In the predictor or independent variable, urban area was coded 0 and rural area was coded 1. 

Data was presented as absolute and relative frequencies, median and interquartile range. Probabilities for low PHTL levels were presented as odds ratio (OR) with 95% confidence interval (CI) and as adjusted OR (ORa). All calculations were performed using SPSS, version 25.0 (IBM Corp., Armonk, NY, USA).

Socio-demographic characteristics included in this analysis were: age (categorised into three groups, from 18 to 29 years, from 30 to 40 years and from 41 to 50 years), gender (women/men), education (university degree—no less than 16 years of education/no-university degree—elementary or secondary school–less than 16 years of education), marital status (living with an intimate partner/living alone), employment (employed/not employed), place of residence (urban/rural), number of children (one, two, three or more) and smoking status (smoker/non-smoker). In addition, the health-related parental and child characteristics included were: breastfeeding (yes/no), self-estimation of health status (average/good/excellent), number of annual visits to a physician (1–2/3–4/5–6/7 or more times) and chronic disease of a child (yes/no).

## 3. Results

### 3.1. Socio-Demographic and Health-Related Characteristics of Respondents

The overall response rate to the survey was 75%. Socio-demographic characteristics of all 432 parents from urban and rural areas are presented in Table 1. Proportions of urban and rural residents were different according to age, education level and employment. These differences were statistically significant. The percentage of parents with ages between 18 and 24 years who were in a rural area were almost five times higher than the percentage of parents with the same ages in the urban area. On the other side, parents, who were between 41 and 50 years, were two times more prevalent in the urban than in rural areas. Most of the parents from the urban area had at least 16 years of education contrary to parents in the rural area. In addition, more parents in urban areas were employed than parents in rural areas. 

After analysing the health-related parental and child characteristics between the two areas, statistically significant differences in breastfeeding and annual visits to pediatrician rate were observed (Table 2). Rates of breastfed children in urban areas were significantly higher than in the rural areas. Parents from rural areas took their children less often to doctors than parents from urban areas. Only 8% of parents from rural and 16% from urban areas took their children to a doctor seven or more times annually. 

### 3.2. Pharmacotherapy Literacy of Respondents

**Figure 1:**
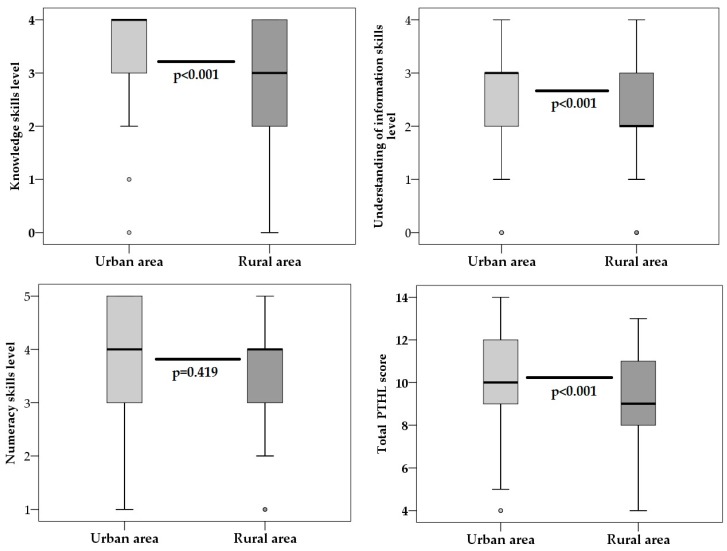
Domains of pharmacotherapy literacy (PHTL) and total PHTL score in urban and rural parents. Median values and interquartile ranges were presented. *p* value was calculated by Mann–Whitney test.

Parent’s PHTL levels expressed as scores per domain (knowledge, understanding information, numeracy) and total PHTL level scores were compared between urban and rural participants. From domains of PHTL, only numeracy skills levels (Z = −0.809; *p* = 0.419) were similar in both groups. Levels of all other domains (knowledge, understanding of information skills and total PHTL), were lower in rural than in urban areas (Figure 1). In the urban area, the median of knowledge skills was 4 and in the rural area it was 3 (Z = −7.39; *p* < 0.001). Median values for understanding of information skills were 3 and 2 in urban and rural areas, respectively (Z = −4.021; *p* < 0.001). Parents in urban and rural areas had median total PHTL scores equal to 10 and 9, respectively (Z = −5.27; *p* < 0.001). Maximal scores for knowledge, understanding of information, numeracy skills level and PHTL level were 4, 4, 5 and 14, respectively. 

**Figure 2:**
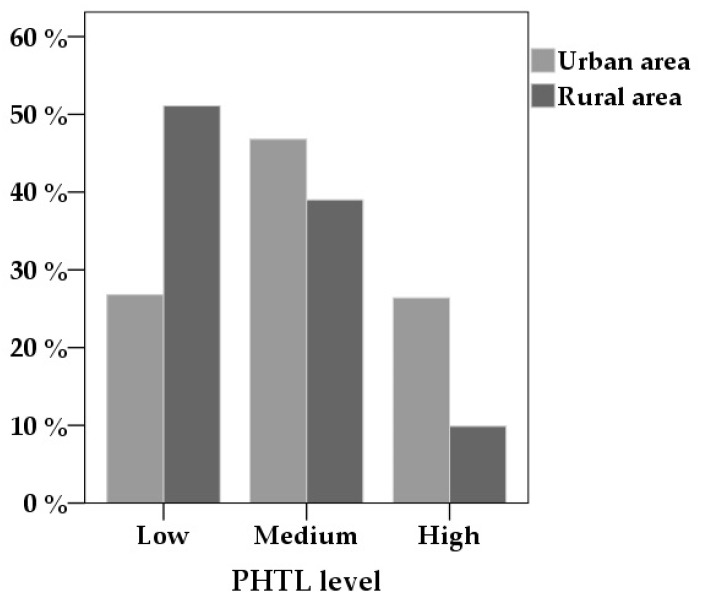
Percent of parents with different pharmacotherapy literacy (PHTL) levels in urban and rural areas.

Every tenth parent (10%) from rural areas and every fourth parent (25%) from urban areas had the highest PHTL level. Contrary to that, around half (51%) and one third (28%) of parents in rural and urban areas, respectively, had low PHTL levels (Figure 2). The difference in PHTL levels between two groups was significant [X^2^(1, *n* = 432) = 33.2; *p* < 0.001].

Effects of living in rural areas on a low PHTL level were examined by logistic regression analysis (Table 3). Results of the regression analyses showed that parents living in rural areas were more likely to have low PHTL levels than parents living in urban areas. With the aim of eliminating the confounding effect of socio-demographic and health-related characteristics on associations between parental living in rural areas and their low PHTL levels, in future analyses we will include all examined variables as independent variables. Low PHTL level of parents was related to living in rural areas independently of their gender, age, number of children, marital status, education level, employment, smoking status, health condition, annual visits to a pediatrician, breastfeeding and chronic disease of a child. 

## 4. Discussion

To our knowledge, this was the first survey to compare levels of pharmacotherapy literacy using a validated PTHL-SR questionnaire [28] among parents from urban and rural areas in Serbia, as well as socio-demographic and health-related characteristics which differed between investigated groups and the influence of living in a rural area on a low PHTL level. In comparison to our previous study [13], we found significant socio-demographic differences among parents of pre-school children living in rural and urban areas. Parents from rural areas were younger and more often had less than 16 years of education compared to parents in urban areas. In addition, more parents from rural areas were unemployed, and less likely to breastfeed children. This was in line with a study conducted in Australia, where health professionals were the main supporters of breastfeeding [30]. Moreover, in rural populations from Serbia, parents are taking their children to the doctor in fewer cases. This finding was similar to the Nigerian demographic health survey study [31] where a rural area was noted for the prevalence of underutilization of children care. 

Regarding levels of pharmacotherapy literacy assessed with a PTHL-SR questionnaire, every tenth parent from a rural area had the highest levels of pharmacotherapy literacy in comparison to parents from an urban area (every fourth parent), while more than half of the parents in rural areas and every third parent in urban areas, respectively, had low PHTL levels. Additionally, levels for all other domains (knowledge and understanding of information) were significantly lower among parents from the rural area. These results were expected because in our previous research on the understanding of information about medicines, the levels of education were an independent predictor of PHTL level [16] and the structure of rural populations with less educated parents could predict such a result. Our results were similar to a study of health literacy in a rural population from Romania, where the population showed inadequate to marginal levels of health literacy within the sample or rural population [24]. 

However, when we examined what could be the reason for low PHTL results, we found that living in a rural area was also an independent predictor for the lowest PHTL results examined by the PTHL-SR questionnaire. The cause of this could have been due to the low availability of health education programs, economic conditions, and cultural background [32]. According to a study of the child health care system of Serbia in 2016 [33], the availability of health educational programs is much higher in urban than in rural areas, where health counseling is significantly more utilized in urban than in rural populations (19.5% vs. 9.1%) [34]. Urgent care is adequate in urban areas, while in rural areas, there is a lack of urgent care use, because of rough roads and bad transportation [33]. Bearing in mind that a pharmacy is not available in every area and within a distance of a few kilometers, parents in rural areas often do not have the possibility to access information about medicines and to ask pharmacists for advice. This is similar to findings of a study from the Scottish Highlands where the respondents who said they did not have a convenient medicine source, regardless of urban/rural classification, did not have easy access to a pharmacy or a public healthcare institution [23]. Moreover, according to a study from Ghana, larger proportions of urban area participants search medicine information from healthcare institutions rather than in rural areas [24]. This was similar to our findings about the number of annual visits to a pediatrician in rural populations, which was significantly lower than in urban populations. More than a half of parents from our study on parental practice in use of OTC (over-the-counter) pediatric medicines highlighted the need for advice from a pharmacist in simple and precise language [11], thus emphasizing that the absence of information could be a risk for low PHTL. 

Pharmacists, as the easiest approachable healthcare providers, could make a contribution in reducing the risk of low PHTL by improving areas of counseling in pharmacy settings among rural populations. These interventions could include easier approaches to pharmacists for the rural population or strengthening educational health programs. 

### Strengths and Limitations

This study provided insight into the PHTL status among parents of pre-school children in rural areas and this could be important as parental practice with medicine use is associated with parent’s level of PHTL. The main limitation of our study was that we did the survey in only one rural municipality of Serbia, due to limited financial resources. Future studies should take into consideration larger populations of people living in rural areas, and in more areas. Another limitation was the use of a cross-sectional study design, which made it difficult to identify causal relationships and to investigate outcomes.

## 5. Conclusions

In our study, every second parent from a rural area had low PHTL levels, which could lead us to conclude that parents of pre-school children who were residents of that rural area had difficulties to obtain, evaluate, calculate, and comprehend basic information about pharmacotherapy necessary to make appropriate medication-related decisions for their child’s safe use of medication. Although, parents from the rural areas were younger, less educated, mostly unemployed and they breastfed children less compared to parents in the urban areas, these characteristics don’t influence the relationship between low PHTL level and living in a rural area. As we showed that a rural area was an independent predictor for the lowest PHTL, our findings are essential to identifying the gaps in parental levels of PHTL, and thus in making effective health education programs for the prevention and management of low PHTL in rural areas.

## Figures and Tables

**Table 1:** Socio-demographic characteristics of urban and rural residents.

Socio-Demographic Characteristics		Urban *n* = 250 *n* (%)	Rural *n* = 182 *n* (%)	Statistics *n* = 432
Gender	Female	207 (82.8)	145 (79.7)	X^2^(1, *n* = 432) = 0.68 *p* = 0.408
	Male	43 (17.2)	37 (20.3)	
Age (years)	18–29	13 (5.2)	45 (24.7)	X^2^(2, *n* = 432) = 38.4 *p* < 0.001
	30–40	184 (73.6)	118 (64.8)	
	41–50	53 (21.2)	19 (10.4)	
Number of children	One child	64 (25.6)	56 (30.8)	X^2^(2, *n* = 432) = 1.87 *p* = 0.392
	Two children	154 (61.6)	108 (59.8)	
	Three children and more	32 (12.8)	18 (9.9)	
Marital status	Living with a partner	232 (92.8)	173 (95.1)	X^2^(1, *n* = 432) = 0.92 *p* = 0.339
	Living without a partner	18 (7.2)	9 (4.9)	
Education	University degree and higher	167 (66.8)	55 (30.2)	X^2^(1, *n* = 432) = 56.4 *p* < 0.001
	No university degree	83 (33.2)	127 (69.8)	
Employment	Employed	230 (92.0)	138 (75.8)	X^2^(1, *n* = 432) = 21.8 *p* < 0.001
	Not employed	20 (8.0)	44 (24.2)	
Smoking	Yes	65 (26.0)	61 (33.5)	X^2^(1, *n* = 432) = 2.88 *p* = 0.090
	No	185 (74.0)	121 (66.5)	

*p* value-according to X^2^ test of independence; Bold *p* values denote statistical significance.

**Table 2:** Parental and child health-related characteristics in urban and rural area.

Health-Related Characteristics		Urban *n* = 250 *n* (%)	Rural *n* = 182 *n* (%)	Statistics *n* = 432
Chronic disease of a child	Yes	32 (12.8)	26 (14.3)	X^2^(1, *n* = 432) = 0.20; *p* = 0.655
	No	218 (87.2)	156 (85.7)	
Breastfeeding of a first child	Yes	225 (90.0)	143 (78.6)	X^2^(1, *n* = 432) = 10.9; *p* < 0.001
	No	25 (10.0)	39 (21.4)	
Annual visits to pediatrician	1–2 times	83 (33.2)	72 (39.6)	X^2^(3, *n* = 432) = 8.09; *p* = 0.044
	3–4 times	75 (30.0)	63 (34.6)	
	5–6 times 7 times and more	52 (20.8)	33 (18.1)	
		40 (16.0)	14 (7.7)	
Parental estimation of health status	Average	36 (14.4)	39 (21.4)	X^2^(2, *n* = 432) = 4.42; *p* = 0.116
	Good	154 (61.6)	109 (59.9)	
	Excellent	60 (24.0)	34 (18.7)	

*p* value-according to X^2^ test of independence. Bold *p* values denote statistical significance.

**Table 3:** Association of living in a rural area with low pharmacotherapy literacy (PHTL) level.

Predictor	β	SE β	Wald X^2^-Square	Df	*p* Value	OR	95% CI
Rural area	1.049	0.206	25.975	1	< 0.001	2.854	1.907–4.273
					*p* value	ORa	95% CI
Rural area *	0.709	0.241	8.635	1	0.003	2.033	1.266–3.262
Goodness of fit test (Hosmer Lemeshow) chi-square = 6.924; df = 8; *p* = 0.545							

* Adjusted by gender, age, number of children, marital status, education level, employment, smoking status, parental health condition, annual visits to a pediatrician, breastfeeding and chronic disease of a child. OR = odds ratio. ORa—adjusted OR; CI = confidence interval; β—unstandardized regression weight; SE—standard error, df—degrees of freedom.
